# Evolution of the hepatitis E virus hypervariable region

**DOI:** 10.1099/vir.0.045351-0

**Published:** 2012-11

**Authors:** Donald B. Smith, Jeff Vanek, Sandeep Ramalingam, Ingolfur Johannessen, Kate Templeton, Peter Simmonds

**Affiliations:** 1 Centre for Immunity, Infection and Evolution, University of Edinburgh, Ashworth Building, King’s Buildings, Edinburgh EH9 3JF, UK; 2 Department of Laboratory Medicine, Royal Infirmary of Edinburgh, Little France, Edinburgh EH16 4SA, UK

## Abstract

The presence of a hypervariable (HVR) region within the genome of hepatitis E virus (HEV) remains unexplained. Previous studies have described the HVR as a proline-rich spacer between flanking functional domains of the ORF1 polyprotein. Others have proposed that the region has no function, that it reflects a hypermutable region of the virus genome, that it is derived from the insertion and evolution of host sequences or that it is subject to positive selection. This study attempts to differentiate between these explanations by documenting the evolutionary processes occurring within the HVR. We have measured the diversity of HVR sequences within acutely infected individuals or amongst sequences derived from epidemiologically linked samples and, surprisingly, find relative homogeneity amongst these datasets. We found no evidence of positive selection for amino acid substitution in the HVR. Through an analysis of published sequences, we conclude that the range of HVR diversity observed within virus genotypes can be explained by the accumulation of substitutions and, to a much lesser extent, through deletions or duplications of this region. All published HVR amino acid sequences display a relative overabundance of proline and serine residues that cannot be explained by a local bias towards cytosine in this part of the genome. Although all published HVRs contain one or more SH3-binding PxxP motifs, this motif does not occur more frequently than would be expected from the proportion of proline residues in these sequences. Taken together, these observations are consistent with the hypothesis that the HVR has a structural role that is dependent upon length and amino acid composition, rather than a specific sequence.

## Introduction

The genome of the single-stranded, positive-sense RNA virus hepatitis E virus (HEV) contains a striking hypervariable region (HVR) that contains multiple substitutions between isolates of the same virus genotype (Tsarev *et al.*, 1992). The HVR occurs within ORF1, which encodes an approximately 1700 residue, 186 kDa polyprotein with a domain structure of NH_2_–methyltransferase, cysteine protease, HVR, ADP-ribose phosphorylase (X or macro domain), helicase, RNA-dependent RNA polymerase–COOH. Analogy with other viruses would suggest that these ORF1 domains are all non-structural proteins, although detailed information about post-translational processing and virion assembly has been difficult to obtain in the absence of an efficient *in vitro* replication system for HEV (Ahmad *et al.*, 2011). Four genotypes of HEV are now recognized by the International Committee on Taxonomy of Viruses, while two distinct isolates from wild boar are currently unclassified; even more divergent viruses isolated from rats (Johne *et al.*, 2010) and chickens (Huang *et al.*, 2004) are also unclassified within the family *Hepeviridae* (Meng *et al.*, 2012).

The function of the HEV HVR is currently unknown. HVRs of other viruses, such as human immunodeficiency virus (HIV) and hepatitis C virus (HCV), are thought to be involved in the escape from host immunological responses during persistent infection. However, as first noted by Tsarev *et al.* (1992), a similar function seems unlikely for the HVR of HEV. This is because the HVR is not expected to be present on the surface of virus particles. In addition, HEV infection is very rarely persistent, with viraemia and faecal shedding usually becoming undetectable by 2 months after infection (Takahashi *et al.*, 2007), except in immunocompromised individuals (Kamar *et al.*, 2012). Furthermore, anti-HEV IgG is protective against reinfection with HEV (Bryan *et al.*, 1994) and antibodies directed against the HEV capsid protein are sufficient to prevent infection (Shrestha *et al.*, 2007). These features suggest that the HVR of HEV does not result from the selection of neutralization-escape mutants.

Several other explanations have been offered for the presence of an HVR in the HEV genome. An early suggestion was that the structure of the genome in this region made it difficult to transcribe, and therefore prone to the introduction of errors during transcription (Gouvea *et al.*, 1998). Another proposal is that the HVR is not required for virus infectivity, as engineered viruses containing deleted forms of the HVR can replicate *in vivo* (albeit with reduced efficiency) and as variants from different genotypes differ so radically (Pudupakam *et al.*, 2009). Alternatively, the proline-rich region might be an integral part or modulator of the protease or helicase domains (Gouvea *et al.*, 1998). SH3 domain-binding motifs are present in the HVRs of all four HEV genotypes, suggesting that these motifs might exploit SH3-mediated interactions to modulate replication or infectivity (Pudupakam *et al.*, 2011) or host range (Purdy *et al.*, 2012). In addition, a variety of functions have been proposed for the HVR and its flanking regions, based upon the presence of conserved linear sequence motifs and from its predicted secondary structure (Purdy *et al.*, 2012). The most commonly proposed function for the HVR is that this region has a structural role as a flexible hinge or spacer between the adjoining ORF1 domains (Koonin *et al.*, 1992). This possibility is supported by a recent analysis of published HVR sequences that reveals that the HVR overlaps an intrinsically disordered region (Purdy *et al.*, 2012).

Two recent papers have added another element to the discussion by describing HEV genomes present in chronically infected patients in which host sequences have been incorporated into the HVR. In one case, a variant containing an in-frame insertion of 171 nt derived from the human ribosomal protein S17 was selected during serial passage of faecal material in HepG2/C3A cells (Shukla *et al.*, 2011). This variant was present in the original sample, although at a level of <1 % of virus genomes. In the second case, the HVR had a 117 nt in-frame insertion derived from the human ribosomal protein S19 gene (Nguyen *et al.*, 2012). This virus represented a substantial minority of the virus present in faecal material and predominated in serum. These observations led the authors to speculate that the divergent sequences of HVR from different HEV genotypes might result from the incorporation and mutation of different host sequences.

The aim of this paper was to investigate these divergent hypotheses by measuring the diversity of the HVR in samples from acutely infected patients and amongst epidemiologically related samples. We have also undertaken a comparative analysis of HEV HVR sequences derived from the large number of complete and partial genome sequences now available.

## Results

### Diversity of HVR in acutely infected individuals

Previous analysis of the HVR of HEV has included comparisons between virus sequences from epidemiologically unlinked infections. These isolates are derived from many different countries, include both human and animal sources, and represent up to six different virus types and an undefined number of subtypes. However, it is not clear whether the extreme variability of the HVR documented in these studies also occurs in acutely infected individuals or following transmission events between individuals or between species. Two recent reports describe HVR variants in chronically infected immunosuppressed patients that contain insertions of host sequences (Shukla *et al.*, 2011; Nguyen *et al.*, 2012), but the generality of these observations is not clear.

In order to study the extent of diversity amongst co-circulating viruses during acute infection, we sequenced HEV HVR PCR products derived from limiting dilution of cDNA from eight patients that were PCR-positive for the ORF2 region (two type 1 and six type 3 virus). A further four patients were PCR-positive for ORF2, but PCR-negative for the HVR. We chose this method of analysis in order to avoid the possibility that sequences derived from cloned amplicons contained substitutions or rearrangements acquired during PCR amplification. In addition, this method avoids the possibility that homogeneity of a sequence dataset reflects the amplification of a single or small numbers of template molecules. Indeed, <10 cDNA templates could be detected in samples from two of the patients; the strategy of sequencing cloned products could have produced many more sequences, but these would still have been derived from only a small number of cDNA templates, so giving a false impression of sequence homogeneity in the virus population. In contrast, the direct analysis of PCR products derived from cDNA templates at limiting dilution will provide an accurate sequence, regardless of PCR misincorporation.

The validity of this approach is illustrated by our observation that all 28 sequences derived from patient 103 were identical (Table 1). This homogeneity was observed despite the fact that these clones were produced using three different combinations of outer primer. Two other patients also infected with genotype 3 virus also showed no variation amongst the molecules sequenced. Limited diversity was observed in the remaining patients, with most virus genomes again being identical within a sample, but with small numbers of substitutions that were found only once in each sequence set. HVR sequences from one individual (patient 21) were more variable. Seventeen sequences were identical apart from three unique substitutions (one synonymous, two non-synonymous). The remaining four sequences differed from the consensus of the first population at 18 positions (13 synonymmous, five non-synonmous) and were identical to each other apart from a single unique substitution. The two virus populations present in this individual (populations A and B) were both of genotype 3, but were as different from each other as they were from virus sequences from different individuals, consistent with this patient being multiply infected. Considering all the patients together, variation of the HVR was extremely limited and there was no clear bias amongst substitutions detected in virus populations from acutely infected patients to either synonymous or non-synonymous changes.

**Table 1.** Limiting-dilution analysis of HVR diversity during acute infection Virus genotype was inferred from the sequence of the ORF2 region of the virus genome.

Patient	Age/sex	Presentingsymptoms	Peak ALT(U l^−1^)	Recent travel history	Virus genotype	No. ofsequencesobtained	Variablesites*	Meandiversity(%)†	Syn‡	Nsyn‡
2	29/M	Jaundice, fever		Indiansubcontinent	1	21	1	0.08	1	0
7	55/F	Jaundice	2989	Spain	3	5	0	0	0	0
101	62/M	Jaundice	2881	None	3	6	0	0	0	0
102	58/M	Jaundice	2245	None	3	20	4	0.20	2	2
103	40/M	Jaundice	6185	Spain	3	28	0	0	0	0
104	25/M	Fulminant hepatitis	4121	India	1	22	5	0.30	2	3
21§	55/M	Jaundice	1993	None	3	21	25	2.6	19	7
					3	A: 17	3	0.14	1	2
					3	B: 4	1	0.40	1	0
22	68/F	Abnormal liver function	4023	Spain	3	18	1	0.20	0	1

* No. of variable sites amongst sequence set.

† Mean *p* distance amongst sequence set.

‡ Syn, no. of synonymous substitutions; Nsyn, no. of non-synonymous substitutions.

§ For patient 21, the diversity amongst the two populations (A and B) of virus sequences detected is also shown separately.

### Variation of HVR in epidemiologically linked samples

As we found no evidence for diversification of the HVR during acute infection, we next surveyed published information to discover whether this region undergoes rapid change during transmission from one individual to another, or when virus passes from one species to another. No HVR substitutions occurred when an immunocompromised individual was infected after blood transfusion (Matsubayashi *et al.*, 2008) or following the transmission of virus from a human to a pig (Bouquet *et al.*, 2012). A single non-synonymous substitution was observed in one of four individuals infected by eating uncooked deer (Takahashi *et al.*, 2004), and a single non-synonymous difference was observed between two individuals that were infected from a common source of food (Miyashita *et al.*, 2012).

Next, in order to investigate the possibility that HVR diversification occurs over a timescale longer than individual transmission events, we surveyed HVR variation amongst sequences that were closely linked on phylogenetic trees of nucleotide sequences (Table 2). Such groups of isolates usually had the same country of origin, although sometimes with a different locality. Differences between HVR sequences amongst closely related sequences were almost entirely due to single-nucleotide substitutions. The only exceptions were a 6 nt insertion/deletion (indel) between two genotype 4 human isolates from Japan (GenBank accession numbers AB291964 and AB253420), and a 3 nt indel in a human isolate from China (HM439284) that grouped with two Chinese pig isolates (EU676172 and DQ279091). For most of these phylogenetic groups, dN : dS was <1 (mean 0.57), the few exceptions being instances where small numbers of very similar sequences were compared (conditions expected to produce noisy estimates of dN : dS). For comparison, analysis of ORF1 for the deer–human transmission (Takahashi *et al.*, 2004) gave a dN : dS ratio of 0.12; when all genotype 3 viruses were compared, the ratio was 0.05. These figures are consistent with the possibility that the HVR is less constrained than other parts of ORF1, but do not provide any evidence for the positive selection of amino acid substitutions in the HVR.

**Table 2.** HVR diversity amongst closely related sequences Sources: Hu, human; Sw, pig; Mo, mongoose; Bo, wild boar.

Sequences	Virus type	No. ofseqs	Country oforigin	Source	HVR mean*p* distance (%)	dN/dS*p* distance
AF010429, AY230202	1	2	Morocco	Hu	1.1	(0 nsyn/1 syn)*
AB291951–57, 60	3	8	Japan	Hu	3.6	0.32
AB443624–26	3	3	Japan	Sw	2.2	0.16
AB074918, -20, AB089824, AB630970	3	4	Japan	Hu	7.3	0.23
AB591733, AB236320	3	2	Japan	Mo	3.7	0.91
FJ426403, -04	3	2	Korea	Sw	1.3	(1 nsyn/0 syn)*
EU495158, -59	3	2	France	Hu	4.5	0.40
EU495171, -48	3	2	France	Hu	1.8	1.80
EU495156, -57	3	2	France	Hu	1.8	0.23
EU495163–66, EU723515–16	3	6	France	Hu	3.5	0.50
EU495178–80	3	3	France	Hu	13.0	0.33
FJ653660, AB369687	3	2	Thailand	Hu	6.9	0.51
AB161717–19 etc.†	4	18	Japan	Hu	2.4	0.38
AB200239 etc.†	4	8	Japan	Hu, Sw	3.1	0.29
DQ450072, GU119961	4	3	China	Sw	9.5	0.93
JF915746 etc.†	4	7	China, Japan	Hu, Sw, Bo	12.8	0.37
AY621103, -06, AY594199	4	3	China	Sw	1.0	1.33
AJ272108 etc.†	4	10	China	Hu, Sw	18.0	0.46
EU366959 etc.†	4	7	China, Korea	Hu, Sw	16.3	0.52

* dN : dS could not be calculated for the sequences with GenBank accession numbers AF010429 and AY230202 (one synonymous substitution only) or for FJ426403 and -04 (one non-syonymous substitution only).

† Group AB161717–19 etc. includes the sequences with GenBank accession numbers AB2209723, -25–29, AB291959, -65–68, AB113311, -12, AB161717 and AB480825; group AB200239 etc. includes AB193176–78, AB097811, -12, AB099347, AB091395, AB220971, AB080575 and AB481227; group JF915746 etc. includes AB602440, AB521806, AB521805, AB602439, AB369690 and EF570133; group AJ272108 etc. includes AY621103, AY621104, AY621106, AY594199, GU361892, AY621105, FJ610232, GU206559 and HM152568; group EU366959 etc. includes GU119960, AB197673, HQ634346, EF077630, AB197674 and FJ763142.

Together, these observations do not provide evidence for selection of amino acid variants of the HVR during acute infection, following individual transmission events or amongst epidemiologically linked viruses.

### HVR evolution within virus genotypes

We have also attempted to understand the evolution of the HEV HVR by comparing the full range of HVR sequences within a virus genotype. Genotype 1 HVR nucleotide sequences were relatively similar to each other (mean distance 0.14, maximum 0.3 amongst 31 sequences) and collinear with no insertions or deletions. A scan of 21 type 1 ORF1 sequences in overlapping 150 nt windows revealed a peak of non-synonymous Jukes–Cantor (J-C) distances at the HVR, but no corresponding increase in synonymous J-C distances (data not shown). Even the most divergent genotype 1 sequences (GenBank accession numbers X98292, AY230202 and AY204877) were related to other type 1 sequences by multiple substitutions rather than by wholesale replacement of the HVR.

**Fig. 1.**
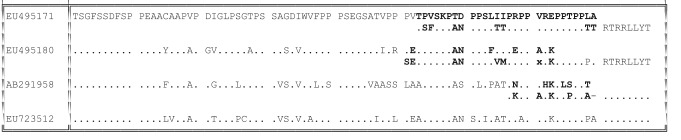
Duplicated regions in genotype 3 HVR sequences. For representative genotype 3 isolates, the HVR amino sequence is shown split into two lines in order to display the proposed duplicated regions (indicated in bold). Dots indicate identity to the top sequence. The last sequence is a representative genotype 3 sequence without a duplicated region.

Comparison of the 153 available sequences of the HVR of genotype 3 viruses revealed them to be more diverse, both in amino acid sequence and, notably, in length. Despite this apparent diversity, these variants were nevertheless related to each other by nucleotide substitution, deletion and duplication. A group of 33 genotype 3f isolates all contained an 87 nt insertion; these comprised 30 human isolates from southern France and three pig isolates from northern Spain. This insertion appears to have arisen as a duplication of the HVR (Fig. 1) (Purdy *et al.*, 2012). Phylogenetic analysis of the 87 nt duplicated region reveals that the 5′ copies from different sequences grouped separately from the 3′ copies in 52 % of bootstrap replications. This is consistent with a single duplication event followed by subsequent independent divergence of the repeats through nucleotide substitution. Other insertions found in genotype 3 sequences appear to have arisen as duplications of 69 nt (GenBank accession numbers EU495178–EU495180) or 39 nt (AB248520) fragments of parts of the same 87 nt region. A further 20 type 3 HVR sequences contained insertions of 12, 15 or 18 nt that were rich in pyrimidines, particularly cytosine, but whose origin was not obvious. A single sequence contained a 3 nt deletion (GenBank accession no. AY115488), while another appeared to derive from three closely spaced deletions of 4, 7 and 4 nt (AF455784). Two unusual HVR sequences isolated from chronically infected patients contained insertions of human-derived sequences (Shukla *et al.*, 2011; Nguyen *et al.*, 2012) that comprised 5–8 % proline. Hence, despite the apparent diversity of type 3 HVR amino acid sequences, all could be derived from other sequences by the processes of duplication, deletion or substitution. The three currently available genotype 3 HVR sequences isolated from rabbits (GenBank accession numbers FJ906895, FJ906896 and GU937805) differed from each other at 31–38 of the 69 amino acid positions, but could still be readily aligned with each other, although not with other genotype 3 sequences.

**Fig. 2.**
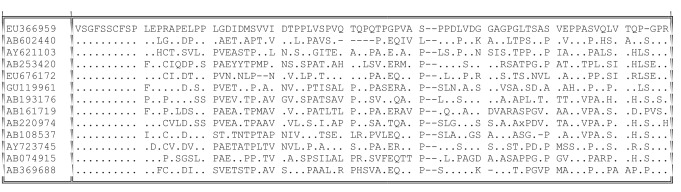
Diversity of HVR in genotype 4. Single representatives of each phylogenetic group of genotype 4 HVR sequences are shown. Identity with the top sequence is indicated by dots; gaps in the sequence are indicated by dashes.

Similar processes can be observed amongst the 69 available genotype 4 HVR sequences. Variants differed by deletions of 3, 6 or 15 nt, the sites of deletion being spread throughout the HVR. Comparison between single representatives of each phylogenetic group of HVR sequences revealed considerable amino acid sequence divergence between sequences (Fig. 2). However, despite this diversity, most differences between these sequences represented amino acid substitutions that were also observed in one or more other type 4 HVR sequences. Of the 34–51 positions that differed between individual pairs of these sequences, only between six and 13 of the differences were unique.

### Common properties of HVR between genotypes

**Fig. 3.**
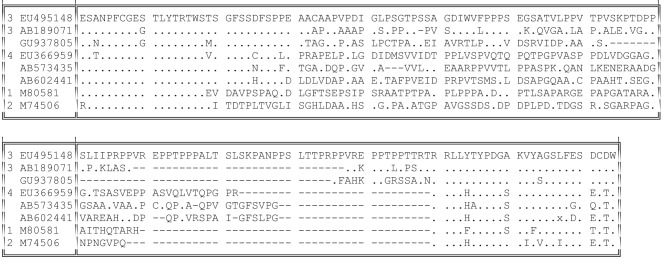
HVR variation between virus genotypes. Representative sequences were genotype 3 (GenBank accession no. EU495148), genotype 3 (AB189071), genotype 3 (rabbit; GU937805), genotype 4 (EU366959), wild boar (AB573435 and AB602441), genotype 1 (M80581) and genotype 2 (M74506). Identity with the top sequence is indicated by dots; gaps are indicated by dashes.

No satisfactory alignment can be made between either the nucleotide or the amino acid sequences of the HVR from different genotypes. Nevertheless, these HVRs shared certain general properties. There was a sharp boundary between the HVR and its well-conserved flanking regions (Purdy *et al.*, 2012); this boundary was shifted by 36 nt towards the 5′ terminus of ORF1 in types 1 and 2, but all had an identical 3′ boundary (Fig. 3) that began with the sequence RRLL in all but one of the 239 sequences available. In consequence of this, genotypes 3 and 4 share a conserved motif immediately preceding their HVR [(T/V)SGFSS(D/C)FSP] that lacks any homology to genotype 1 or 2 sequences at the equivalent position.

**Fig. 4.**
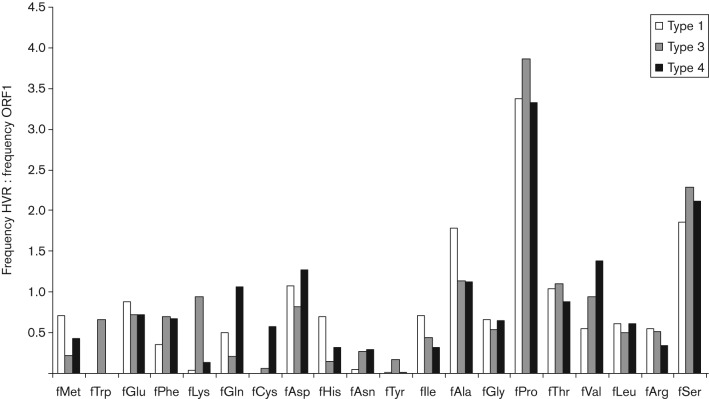
Biased amino acid composition of the HVR. The ratio of the mean amino acid composition of HVR of genotypes 1, 3 and 4 compared with that for ORF1 as a whole is shown for each amino acid.

All of the HVRs studied here share the property of being rich in proline. We sought to describe the amino acid composition of the HVR in more detail by comparing amino acid frequencies with those observed in ORF1 as a whole (Fig. 4). For genotypes 1, 3 and 4, the amino acid composition of the HVR differed from that of the remainder of ORF1 in that there was a relative excess of proline (means of 24–30 % compared with 7–8 % for ORF1 as a whole) and serine (means of 11–14 % compared with 6 % for ORF1). Similar excesses of proline and serine were observed for the single HVR sequences available for genotype 2, for the divergent viruses isolated from wild boar and for the three rabbit HEVs that are related most closely to genotype 3. The HVRs of all genotypes were also consistently deficient in leucine, arginine and tyrosine residues.

The more distantly related rat and avian HEV genomes also contain a proline-rich section at a similar position in their genomes. In the case of rat HEV, this comprised 86 codons containing 28–29 % proline, although this region is not notably variable between the two sequences available. In contrast, a 75–77 residue region of avian HEV contained 23–27 % proline and was highly divergent between different the five sequences available.

**Fig. 5.**
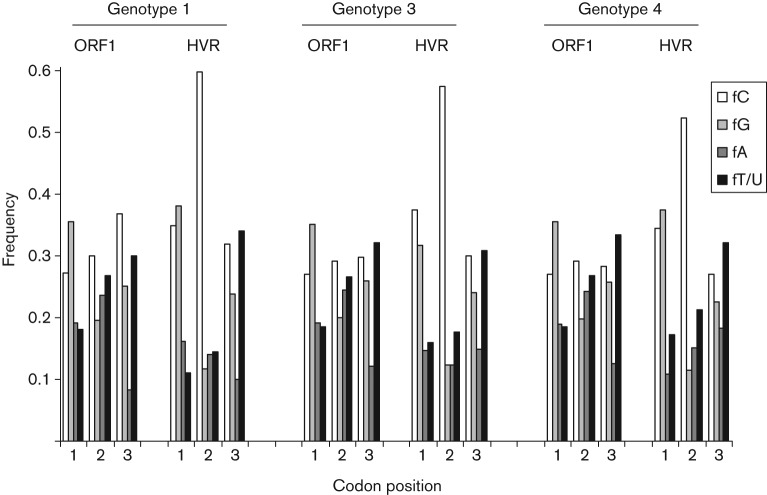
Over-representation of cytosine at the second codon position in the HVR. For the first, second and third position of codons, the frequency of each nucleotide in ORF1 as a whole or in the HVR is shown for genotypes 1, 3 and 4.

One explanation for the biased amino acid composition of HVR might be that this region was relatively rich in cytosine (mean of 38–42 % compared with 28–31 % for ORF1 as a whole for types 1, 3 and 4). The presence of polycytidine has been noted in some genotype 3 viruses (Erker *et al.*, 1999). The high frequency of proline residues, encoded by CCN codons, might then be a consequence of a local bias towards cytosine in this part of the genome. However, analysis of the frequency of cytosine at different codon positions reveals that, compared with ORF1 as a whole, cytosine was abnormally abundant only at second codon positions in the HVR, where the frequency rose to between 50 and 60 % (Fig. 5). A similar comparison of the proline- and serine-rich HVRs of 62 alphavirus nsP3 sequences also revealed a bias towards cytosine at second codon positions, although this bias was less extreme than that observed for HEV (data not shown). This suggests that cytosine is abundant in the HVR of HEV because of the increased frequency of the amino acids proline and serine, which have cytosine at the second position.

**Fig. 6.**
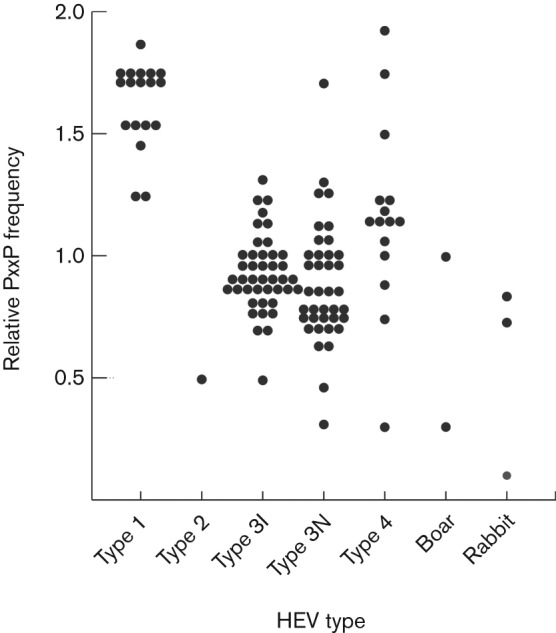
PxxP motifs occur at a level expected by chance in the HVR. For each genotype, the figure shows the ratio of the number of PxxP motifs in the HVR compared with the number expected by chance for a peptide of the same amino acid composition and length. Sequences comprised epidemiologically unlinked sequences from genotype 1 (*n* = 18), genotype 2 (*n* = 1), genotype 3 sequences with (type 3I, *n* = 42) and without (type 3N, *n* = 38) a duplicated region, genotype 4 (*n* = 15) and divergent types from wild boar (*n* = 2) and genotype 3 sequences from rabbits (*n* = 3).

Another characteristic of the HVR that is shared between HEV genotypes is the presence of SH3-binding motifs such as PxxP (Pudupakam *et al.*, 2011). SH3 domains have previously been reported to bind to HEV ORF3 (Korkaya *et al.*, 2001), which contains 18–20 % proline and contains the well-conserved sequence PSAPPLPP. More detailed analysis of the HVR sequence set described here reveals that, with the exception of the rabbit-derived sequence with GenBank accession no. FJ906896, all HEV HVRs contained between one and seven PxxP motifs. However, as the HVR is proline-rich, these motifs might also arise by chance. In order to test this possibility, we constructed a perl program to estimate the number of PxxP motifs expected in an amino acid sequence with a given amino acid composition and length. The results of this analysis for a dataset comprising epidemiologically unlinked sequences from different genotype or subgroupings are shown in Fig. 6. Whilst an excess of PxxP motifs over the number expected was observed for all genotype 1 HVR sequences and for most genotype 4 HVR sequences, fewer PxxP motifs than expected were observed for the single genotype 2 HVR and on average for genotype 3 sequences, whether or not they contained an internal duplication (0.93 for insert, 0.87 for non-insert). Similarly, no excess of PxxP motifs was observed for the three rabbit sequences or the two divergent genotypes isolated from wild boar. This analysis suggests that any polypeptide with the same proline composition of the HEV HVR will have a similar number of PxxP motifs.

## Discussion

We describe here an analysis of sequence variation and evolution of the HEV HVR in acutely infected individuals, amongst epidemiologically or phylogenetically linked isolates, and within and between virus genotypes. Despite the considerable variation observed between HVRs from different genotypes and even within genotypes, very little variation was observed amongst variants co-circulating within acutely infected individuals. In addition, no bias towards non-synonymous substitutions was observed amongst these datasets. Similar conservation of the HVR has recently been described in a high-density sequencing study of virus from an acutely infected HEV patient (Bouquet *et al.*, 2012), although in this case the number of viral sequences in the population that were actually sampled was unknown and probably lower than the number of sequences characterized. Variation was also restricted amongst sequences within a transmission network and amongst phylogenetic groupings of HVR sequences. In all of these groupings, there was no evidence for an excess of non-synonymous substitutions over synonymous substitutions that would suggest adaptive evolution or immune escape from B- or T-cell responses.

There is, however, more consistent evidence of a bias towards non-synonymous substitutions during the passage of HEV. For example, three substitutions, all non-synonymous, were observed in the HVR of a second passage of human type 1 virus in a rhesus monkey (Arankalle *et al.*, 1999). Cell-culture passage of a type 3 virus in AF549 cells resulted in no changes in the HVR, whilst virus passaged in PLC/PRF/5 cells accumulated one non-synonymous change and, in a separate experiment, the same non-synonymous change and two synonymous substitutions (Lorenzo *et al.*, 2008). Five non-synonymous and two synonymous substitutions and the insertion of a host sequence differentiate virus from a chronically infected individual and after passage in cell culture (Shukla *et al.*, 2011), although these substitutions may have been pre-existing in the inoculum. Finally, virus from another chronically infected patient and containing a host-derived insertion in the HVR appeared to be unstable, with a variety of deleted forms appearing after passage (Nguyen *et al.*, 2012).

A recent study used the one-rate fixed effects likelihood method (fel) in HyPhy (Pond *et al.*, 2005) to provide evidence of positive selection at a total of four, five or ten codons within the HVR in genotypes 1, 3 and 4, respectively (Purdy *et al.*, 2012). However, analysis of the same genotype 1 and genotype 4 datasets using other tests for positive selection within the HyPhy suite (two-rate fel, meme, slac, rel and fubar) failed to identify some or all of these sites (data not shown).

Our comparison of HVR diversity within different virus genotypes is consistent with HVR evolution occurring through the processes of substitution and duplication/deletion within the HVR. No evidence was obtained among the variants characterized in the current study for the acquisition and incorporation of exogenous sequences into the HVR by non-homologous recombination. The HVRs of all genotypes shared the property of being relatively rich in proline and serine residues, giving rise to, rather than being a consequence of, an increased frequency of cytosine in this part of the genome. We have not found consistent evidence of positive selection at any amino acid site within the HVR.

These observations are relevant to the evaluation of the various hypotheses advanced in the literature as to the function of the HVR in virus replication. An early suggestion that the HVR might simply be the result of error-prone replication (Gouvea *et al.*, 1998) seems unlikely, given the consistent biases in amino acid composition (Fig. 4) and the codon position-specific distortion in nucleotide frequency (Fig. 5). In addition, we observed a peak in non-synonymous distances but not of synonymous distances at the HVR in a sliding-window comparison of type 1 ORF1 sequences. These observations imply that the presence of amino acid substitutions in the HVR is not the result of hypermutation in this part of the genome.

Others have interpreted the lack of identity and extensive length variation between HVR sequences from different genotypes as evidence that the HVR is not essential for virus replication (Pudupakam *et al.*, 2009). However, there are several lines of evidence that the HVR does have a biological function. Firstly, a proline- and serine-rich HVR is present at a similar location in all published HEV sequences in both human- and pig-derived isolates and in the more divergent rat and avian HEV genomes. This region is hypervariable in the avian sequences but not the rat sequences, although this may reflect the paucity of sequence data currently available for rat variants. A proline-rich region is also present in the more distantly related cutthroat trout virus (Batts *et al.*, 2011). Secondly, *in vitro* experiments suggest that complete deletion of the HVR of type 1 or type 3 virus impairs the ability of virus to replicate in transfected cells or intrahepatically infected pigs (Pudupakam *et al.*, 2009).

The simplest potential function for the HVR would be as an inert spacer or hinge between two functional domains (Koonin *et al.*, 1992). Relevant to this possibility is the suggestion that three type 1 HVRs share a common hydropathy profile (Tsarev *et al.*, 1992), although this conclusion is difficult to quantify. More convincingly, a recent report suggests that the HVR is part of a larger region that is intrinsically disordered (Purdy *et al.*, 2012). Such a passive role for the HVR is consistent with the extensive length variation observed between HVRs of different genotypes (and within genotype 3) and by the lack of sequence identity between HVRs from different genotypes. However, it would appear that there is a lower limit to such length variation, as artificial constructs containing a series of deletions of the HVR display luciferase activity approximately in proportion to the length of HVR remaining (Pudupakam *et al.*, 2011). Similar features have been described for the env protein of type C retroviruses, which contains a C-terminal unstructured 40–49 residue HVR with an excess of proline (>30 %) and serine relative to the rest of env. This HVR is preceded by a 15 residue proline-rich region that is well-conserved within Moloney murine leukemia virus (MuLV) isolates. Deletions or insertions within the MuLV env protein HVR do not affect virus growth (Ott *et al.*, 1990; Kayman *et al.*, 1999), and it has been suggested that the overall amino acid composition of this region is important for the processing of env into two subunits and for their subsequent interaction (Wu *et al.*, 1998). Parenthetically, a similar function as an inert spacer might be proposed for the 31 aa insertion in genotype 3 isolates from rabbits, which occurs close to the junction between the ADP-ribose phosphorylase (X or macro domain) and the helicase domains. This insertion also contains an excess of proline residues relative to the rest of ORF1. The more distantly related rat HEV genome contains an insertion in a similar position, containing 24 % proline.

The possibility that the HVR of HEV is the target of neutralizing immune responses arises by analogy with the HVRs of HCV and HIV. Another example is the proline-rich HVR of the MSA1 and MSA2 proteins of the intracellular parasite *Babesia bovis*, which may play a role in immune-mediated escape (Berens *et al.*, 2005; LeRoith *et al.*, 2006). The HVR of HEV does appear to be immunogenic, as an antigen derived from the HVR of a genotype 1 virus had a sensitivity of 75 % against a serological panel comprising individuals infected with types 1, 3 and 4 (Osterman *et al.*, 2012). However, we found no consistent evidence for positive selection of HVR variants in our analysis of HVR variation in acutely infected individuals (Table 1), upon transmission between humans, from animals to humans, or amongst epidemiologically linked sequences (Table 2). We were unable to study earlier or later samples from the acutely infected patients reported here, and it remains possible that virus diversification occurs during the 1–2 months of viraemia. However, in this case one might expect to see diversity within virus populations, yet the diversity within infected individuals was very restricted (mean distances of 0–0.004 %), apart from one case in which the individual appeared to be multiply infected (Table 1). Hence, the timescale over which HVR substitution occurs appears to be longer than that of single transmission events, even if these are between different species or during defined outbreaks.

Specific functions for the HVR have also been proposed based on the presence of linear motifs, possibly with a role in the shuttling of virus between different hosts (Purdy *et al.*, 2012). However, our analysis provides no evidence for specific alteration of the HVR following transmission between different host species (Table 2) and we have been unable to detect any phylogenetic segregation of human and non-human HVR sequences (data not shown). Hence, it does not appear that the HVR contains determinants of host range. PxxP motifs within the HVR might represent SH3-binding domains (Pudupakam *et al.*, 2011), as demonstrated for HEV ORF-3 (Korkaya *et al.*, 2001), the alphavirus nsP3 HVR (Neuvonen *et al.*, 2011) and the P150 replicase protein of rubella (Suppiah *et al.*, 2012). Although these motifs are present in all but one of currently described HVR sequences, they would be expected to arise at a similar frequency by chance in any similarly proline-rich region. This suggests that the presence of PxxP motifs is a consequence of the proline content of the HVR; whether some or all of these motifs are actually SH3-binding domains requires experimental proof.

A variety of linear motifs have been identified associated with the HVR (Purdy *et al.*, 2012), but these all occur in the conserved regions flanking the HVR rather than the HVR itself. A number of very general functions (enzymic activity, ligand, nucleotide binding, catalysis, ion binding) have been proposed for the HVR based upon the predicted secondary structure of a genotype 3 isolate (Purdy *et al.*, 2012). Peptides containing a high proportion of proline are often ligands, as the cyclized side-chain restricts movement of the backbone (Williamson, 1994; Kay *et al.*, 2000), but we have been unable to identify motifs that are conserved amongst the divergent HVR sequences of different genotypes.

Finally, the suggestion has been made that divergent HVR sequences might represent evolved host-derived sequences acquired during chronic infection (Shukla *et al.*, 2011; Nguyen *et al.*, 2012). A blast search of GenBank with representatives of types 1, 2, 3 and 4 failed to reveal any significant matches with host sequences (data not shown), although homology with host sequences could have been masked by subsequent adaptive evolution. A more convincing argument against HVR diversity being host-derived comes from our comparison of epidemiologically unrelated sequences of genotypes 1, 3 and 4, which revealed that these sequence groups were related to each other by the processes of substitution, duplication and deletion.

In conclusion, our analysis of HEV HVR evolution and variation suggests that the HVR is important for virus replication and may have a structural rather than a regulatory or enzymic function. More detailed investigation of the processing and structure of ORF1 proteins in *in vitro* systems will be required in order to define the role of the HVR in HEV replication and pathogenesis. In this regard, the dissection of HVR function may be facilitated by the availability of HVRs from different virus genotypes with dissimilar amino acid sequences, but presumably sharing common functionality. For example, the specificity of host-cell proteins binding to artificially expressed fusion proteins containing the HVR could be assessed by comparison with binding to a divergent HVR from a different genotype.

## Methods

### 

#### Clinical samples.

Serum samples from 17 individuals presenting at the Royal Edinburgh Infirmary between April 2006 and April 2012 with acute hepatitis and serological evidence of recent HEV infection were investigated for viraemia by PCR. Virus RNA was extracted from 140 µl serum using a QIAamp viral RNA mini kit (Qiagen) following the manufacturer’s protocol, and eluted in a volume of 60 µl elution buffer. HEV RNA was then amplified using the Access RT-PCR system (Promega) in 50 µl reactions containing 10 µl RNA and the ORF2 primers 5′-CARGGYTGGCGYTCBGTYGAGAC-3′ (outer sense) and 5′-CCYTTRTCCTGCTGVGCRTTCTC-3′ (outer antisense). Reactions were incubated at 48 °C for 45 min, followed by 3 min at 94 °C and then 30 cycles of 94 °C for 18 s, 55 °C for 21 s and 72 °C for 90 s. A second round of PCR was carried out using 1 µl of the primary reaction in a 20 µl reaction containing Go *Taq* (Promega) and the primers 5′-TAYACYAAYACRCCTTAYACYGGTGC-3′(inner sense) and 5′-GTCGGCTCGCCATTGGCYGAGACGAC-3′ (inner antisense); cycling parameters were 30 cycles of 92 °C for 18 s, 55 °C for 21 s and 72 °C for 90 s.

#### Nucleotide sequencing and phylogenetic analysis.

Nucleotide sequences of amplicons were obtained on both strands using a BigDye Terminator Cycle Sequencing kit (Applied Biosystems) and analysed on an ABI 3730 machine operated by Genepool (University of Edinburgh). Viruses were typed by phylogenetic analysis of sequences against HEV sequences available in GenBank using mega 4 (Tamura *et al.*, 2007). GenBank accession numbers for the sequences described here are JX270834–JX270947.

#### Sequence analysis of HVR at limiting dilution.

cDNA was generated using either random hexamer or the appropriate outer antisense primer in 20 µl reactions containing 5 mM MgCl_2_, 1 mM dNTPs, 20 U RNasin (Promega), 1× RT Buffer (Promega) and 7 U avian myeloblastosis virus reverse transcriptase (Promega). The resulting cDNA was then diluted until a minority of replicate reactions resulted in a product. PCR amplification of cDNA used Go *Taq* DNA polymerase (Promega) and type-specific primer sets as follows: type 3: outer sense 301, 5′-TRTGGYTRCAYCCYGAGGG-3′, or 302, 5′-GGRCAYMTYTGGGAGTCTGC-3′; outer antisense 354, 5′-GTYTCNCGRTAYGCYGCCTCNARCCTC-3′, or 352, 5′-CCARTCACATRCYGAYTCRAACA-3′; followed by inner sense 304, 5′-ACHYTKTAYACYCGNACYTGGTC-3′, and inner antisense, 5′-CCYGCRTANACCTTRGCVCCRTC-3′. Type 1: outer sense 101, 5′-TTGCCCCCGGYGTTTCACCCCGGTC-3′, outer antisense 152, 5′-TGGTCAACATTAGACGCGTTAAC-3′; followed by inner sense 104, 5′-ACACTTTACACCCGYACTTGGTCGGA-3′, and inner antisense 151, 5′-AAYACCTTAGAGCCATCCGGGTAGGT-3′. Cycling parameters were 30 cycles of 92 °C for 18 s, 50 °C for 21 s and 72 °C for 90 s. PCR products from individual reactions were sequenced as described above.

#### Sequence collection.

A total of 6060 HEV sequences were downloaded from GenBank on 13 March 2012 and aligned using sse v. 1.0 (Simmonds, 2012). From this collection, we identified HVR sequences amongst 180 unique complete genome sequences and 58 additional partial genome sequences, comprising in total 29 type 1, one type 2, 145 type 3 and 61 type 4 sequences, and two unclassified sequences isolated from wild boar.

#### Calculation of expected PxxP frequency.

The frequency of PxxP motifs observed in 1000 sequences of the same length and amino acid composition as a test sequence was calculated using a program written in perl (available upon request from the authors).
